# Strengthening Patient Safety in Respiratory Wards by Developing a Weekend Continuity Checklist

**DOI:** 10.7759/cureus.97546

**Published:** 2025-11-23

**Authors:** Zoha Iftikhar

**Affiliations:** 1 Medical Education, East Kent Hospitals University NHS Foundation Trust, Ashford, GBR

**Keywords:** continuity of care, handover, nhs hospitals, patient safety, weekend shifts

## Abstract

Background: Effective clinical handover is essential for continuity and patient safety, particularly during weekends when staffing levels are reduced. Incomplete or unclear handovers contribute to delays, missed investigations, and preventable errors. This quality improvement project aimed to enhance the quality and reliability of weekend handovers on a respiratory ward in a UK District General Hospital.

Methods: Over an eight-week period, the project employed two Plan-Do-Study-Act cycles to assess and improve handover practices. Baseline data from 44 weekend handovers were audited for completeness of blood test requests, antibiotic prescriptions, and discharge documentation. A structured weekend continuity checklist was then introduced, prompting weekday teams to document key patient information before the weekend. Post-intervention data were collected from 50 weekend handovers and compared using chi-square or Fisher’s exact tests.

Results: Baseline data revealed deficiencies in blood test handovers (20%, 9/44 incomplete), antibiotic coverage (11%, 4/38 missed), and discharge summaries (55%, 6/11 incomplete). Following implementation of the checklist, missed blood test handovers decreased to 2% (1/50, p = 0.024), antibiotic lapses reduced to 4% (2/48, p = 0.469), and all discharge summaries were completed (100%, p = 0.038). Venous thromboembolism assessments and escalation plans were documented for all patients post-intervention, reflecting improved preparedness and communication.

Conclusion: Introducing a structured weekend handover checklist significantly improved handover completeness, documentation quality, and clinical preparedness for weekend teams. This low-cost, sustainable tool reduced process-related errors and enhanced patient safety and continuity of care. The findings support wider adoption of standardized handover protocols to strengthen weekend care delivery across hospital settings.

## Introduction

Handover, the process of transferring responsibility for patient care between healthcare professionals, is a critical component of safe and effective clinical practice. The General Medical Council’s Good Medical Practice emphasizes that every doctor must ensure effective handover to maintain continuity of care [1]. The Royal College of Physicians notes that communication failures during handover can lead to missed diagnoses, unnecessary investigations, and inappropriate treatment [2]. Recent studies have reinforced the need for structured, standardized handovers to reduce clinical risk and improve outcomes, particularly during weekend and out-of-hours periods [3-5]. Modern frameworks such as the I-PASS system have demonstrated sustained improvements in communication reliability [6], while initiatives like "Modernising the Weekend Medical Handover" highlight the ongoing need to optimize information transfer across shifts [7].

Despite these advances, handover remains a vulnerable stage in patient care. National data suggest higher inpatient mortality over weekends, partly attributed to inadequate communication between weekday and weekend teams [3]. This underscores the need for practical, locally adaptable solutions to strengthen handover processes.

This quality improvement (QI) project was conducted in William Harvey Hospital, which is a district general hospital in the UK. The hospital uses an electronic handover system designed for interdepartmental referrals, which also supports weekend handovers via a structured situation-background-assessment-recommendation format. However, informal feedback from clinical staff revealed several usability challenges: frequent sign-ins disrupted workflow, leading to missed or incomplete updates, particularly during busy weekday shifts. These issues are compounded by weekend staffing patterns-one senior house officer covering multiple wards and a single registrar overseeing all medical and geriatric units-creating additional barriers to safe and efficient handover [4,5].

The primary objective of this project was to improve the efficiency, completeness, and reliability of weekend handovers in the respiratory wards by optimizing the handover system and promoting standardization among junior doctors.

## Materials and methods

Problems and aims

Problem Statement

An initial assessment in the respiratory ward identified significant deficiencies in weekend handover processes. Essential management information was inconsistently communicated from weekday to weekend teams, leading to gaps in care such as missed investigations, unprescribed medications, and interruptions in continuity. These issues were particularly concerning for complex respiratory cases, such as empyema, pleural effusion, and chronic obstructive pulmonary disease (COPD), which require frequent review and careful titration of therapies such as noninvasive ventilation (NIV).

The weekend medical team comprised only one junior doctor with limited senior support, emphasizing the need for structured, reliable weekend management plans to ensure patient safety and consistent care delivery.

Aims

The primary objective of this quality improvement project (QIP) was to determine whether introducing a structured weekend handover checklist, implemented through iterative Plan-Do-Study-Act (PDSA) cycles, could improve the quality and safety of weekend patient care in the respiratory wards.

Specifically, the project aimed to 1) increase the proportion of patients with complete weekend handover documentation (including diagnosis, current plan, and weekend task list) from baseline to ≥90% within four weeks following the introduction of the handover checklist and 2) ensure that all patients requiring weekend review (e.g., those on NIV or awaiting investigations) had a documented escalation status or monitoring plan prior to handover.

Methods

This prospective quality improvement project was conducted over eight weeks in the respiratory ward of a district general hospital in the United Kingdom. The project aimed to address gaps in the completeness and reliability of weekend handovers. Despite the use of an electronic handover system, weekend handovers were often incomplete or inconsistently updated, leaving doctors managing unfamiliar patients without clear continuity plans. All adult inpatients under the respiratory team with scheduled weekend handovers were included. Patients discharged before the weekend or receiving only palliative care were excluded.

PDSA Cycles

The project followed the Plan-Do-Study-Act (PDSA) framework (Institute for Healthcare Improvement, 2023) [7], with two iterative cycles aimed at improving the weekend handover process.

Cycle 1: Cycle 1 involves the following: Plan: identify deficiencies in the existing electronic handover system. Baseline observations and informal staff feedback were used to determine gaps in documentation and escalation planning; Do: conducted an audit of weekend handovers to evaluate completeness and clarity. Informal staff input was collected regarding practical barriers to effective communication; Study: analysis revealed recurring issues, including incomplete escalation plans, inconsistent documentation of pending tasks, and variability in handover quality between clinicians; Act: developed a structured weekend continuity checklist (see the Appendix) to standardize documentation of patient background, escalation status, pending investigations, and anticipated weekend needs.

Cycle 2: Cycle 2 involves the following: Plan: introduce the checklist for patients requiring weekend review; Do: the checklist was implemented each Friday by weekday teams. Staff were instructed on its use, and its integration into routine handover was observed; Study: a repeat audit was conducted four weeks post-implementation to evaluate improvements in completeness of documentation and inclusion of escalation plans. Compliance with checklist use was monitored by recording the proportion of eligible patients for whom the checklist was completed; Act: findings were used to provide feedback to staff and inform minor adjustments to the checklist workflow to enhance usability and adherence.

Tools and Checklists

The structured weekend continuity checklist was developed specifically for this project. It prompted documentation of essential clinical information, including patient identifiers, background, escalation status, pending investigations, and anticipated weekend needs. Where applicable, other tools such as venous thromboembolism (VTE) risk assessments were completed using standard National Institute for Health and Care Excellence guidance 2018 [9]. All tools were publicly available or original to the project.

Data Collection and Analysis

Baseline and post-intervention measurements were collected for each patient on parameters including blood tests requested, antibiotics prescribed, discharge summaries completed, VTE assessments, and documented management plans. In total, 44 patient handovers were assessed during PDSA Cycle 1 (baseline) and 50 during PDSA Cycle 2 (post-intervention).

Patients were eligible for inclusion if they were expected to remain in the hospital over the weekend and required ongoing medical review or intervention. Patients discharged before Friday or receiving only palliative care were excluded.

For quantitative comparisons between baseline and post-intervention data, categorical variables were analyzed using chi-square tests or Fisher’s exact tests, depending on expected counts. Test statistics (χ²) and p values are reported where appropriate (Table 1). Parameters without baseline data were reported descriptively, and no statistical comparisons were performed for these variables.

**Table 1:** Comparison of weekend handover parameters before and after implementation of the continuity checklist (PDSA cycle 1 vs. cycle 2), including statistical analysis Comparisons are between baseline (PDSA Cycle 1) and post-intervention (PDSA Cycle 2). The project followed the PDSA quality improvement framework, a freely available model for iterative healthcare improvement [8] ^*^Chi-square test (with Yates Correction) was used where expected counts ≥5; for chi-square tests, the statistic and degrees of freedom are reported as χ². Analyses were performed using GraphPad (publicly accessible online statistical software; Dotmatics, Boston, Massachusetts) ^**^Fisher’s exact test was used otherwise. Analyses were performed using GraphPad PDSA: Plan-Do-Study-Act, VTE: venous thromboembolism; N/A: not available

Parameter	Baseline (first PDSA cycle)	Post-intervention (second PDSA cycle)	Improvement (%)	Test used	Test statistic	p value
Blood tests not requested	20% (9/44 patients)	2% (1/50 patients)	90%	Chi-square test^*^	χ² ≈ 9.2^*^	0.024
Antibiotics requiring represcription	11% (4/38 patients)	4% (2/48 patients)	64%	Fisher’s exact test^**^	N/A	0.469
Incomplete discharge summaries	55% (6/11 patients)	0% (0/7 patients)	100%	Fisher’s exact test^**^	N/A	0.038
VTE assessments completed	Not measured	100% completion	N/A	N/A	N/A	N/A
Clear plans in place (IV fluids, nutrition, respiratory-specific care, deterioration management)	Not measured	100% completion	N/A	N/A	N/A	N/A

Ethical Considerations

This project was registered with and approved by the Quality Improvement (QI) Team, East Kent Hospitals University National Health Service (NHS) Foundation Trust. The QI Team reviewed the proposal and confirmed that it met the criteria for a service evaluation and quality improvement activity; therefore, formal Research Ethics Committee approval was not required. No experimental procedures or randomization were involved, and all data were anonymized in accordance with institutional and NHS data protection policies.

*Disclosures*

No external funding was received for this work. The author declares no competing interests.

## Results

Baseline data

Baseline data were collected from a total of 44 handovers on the respiratory ward of a district general hospital over a four-week period. Data were recorded every Friday after 18:00, once the weekday team had completed routine tasks and prepared weekend handovers for the on-call team. The aim of this baseline audit was to identify the frequency and nature of incomplete or inadequately communicated handover tasks before introducing the intervention.

Three key parameters were monitored during this period: 1) Blood test requests: whether blood tests were ordered and clearly handed over for weekend review; 2) antibiotic prescriptions: whether ongoing antibiotic courses were prescribed on the electronic prescribing and medicines administration system to ensure continuous cover over the weekend; and 3) discharge summaries: whether discharge documentation was completed for patients with a documented plan for discharge before 09:00 on Monday morning.

Analysis of the baseline data revealed several deficiencies in the existing handover process. Over the four-week period 1) 20% (9/44) of eligible patients had no blood tests requested or no clear handover for reviewing blood results; 2) 11% (4/38) of patients had antibiotics due to running out over the weekend and required represcription by the weekend doctor; and 3) 55% (6/11) of discharge summaries for patients identified as potential weekend discharges were incomplete and left for the weekend team to finalize.

These findings highlighted consistent communication gaps and incomplete task transfers between the weekday and weekend teams, reinforcing the need for a structured and standardized approach to weekend handovers to improve continuity of care and reduce avoidable workload for on-call staff.

Post-intervention data

Following the implementation of the structured weekend handover checklist, data were collected over a four-week period to evaluate its impact on handover completeness and patient management. The same parameters assessed during the baseline audit were reevaluated, incorporating VTE assessments [9] and clearer management plans to capture improvements in documentation and planning.

A total of 50 handover events were reviewed during this cycle. The post-intervention audit demonstrated marked improvements across all key parameters compared with baseline, particularly in the completeness of blood test handovers and discharge summaries (both statistically significant; p < 0.05), as shown in Table 1. Compliance with VTE assessments and documentation of clear, legible management plans reached 100%, while missed antibiotic prescriptions showed a nonsignificant reduction.

Overall, the structured checklist led to measurable improvements in continuity of care, communication, and weekend preparedness, reducing the risk of omissions and improving the reliability of handovers between weekday and weekend teams. Baseline and post-intervention results were compared, demonstrating clear improvement in handover completeness (Table 1).

## Discussion

Weekend hospital care has long been recognized as a vulnerable period for patient safety. Studies such as Freemantle et al. have shown that patients admitted or cared for over the weekend face higher risks of complications and mortality, often due to incomplete handovers and reduced senior staffing [3]. In our respiratory ward, weekend teams frequently managed unfamiliar patients with complex respiratory conditions such as COPD, empyema, and pleural effusion, with limited guidance from weekday staff.

This project demonstrates that a small, structured intervention, a weekend handover checklist, can meaningfully improve the completeness and reliability of handovers. By ensuring that pending investigations, escalation plans, and anticipated weekend needs were clearly documented, the checklist enabled weekend clinicians to work more safely and efficiently. These results align with several published QI initiatives that emphasize structured communication and standardization as key drivers of safer handover [10-14].

Our findings are consistent with prior work by Palmer et al. and Michael and Patel, who demonstrated that simple checklist-based approaches (e.g., the FRIDAYS framework) improved documentation of essential weekend tasks and reduced omission errors [4,10]. Similarly, Till et al., and Mehra and Henein reported that integrating user-centered, standardized templates into routine workflow enhanced clarity and accountability during shift changes [11,12]. In our project, comparable gains were observed in blood test requests and discharge planning, both of which improved significantly following checklist implementation (p < 0.05).

Importantly, while the statistical improvements were notable, their clinical significance lies in process reliability rather than direct patient outcomes. Reducing the proportion of incomplete handovers directly decreases the risk of missed investigations or delayed interventions, both key contributors to weekend morbidity [15,16]. Although we did not measure patient-level outcomes such as readmission or length of stay, the observed improvements in task completion and documentation represent meaningful progress toward safer, more consistent weekend care.

Qualitative staff feedback, although informal, suggested the checklist increased confidence, consistency, and task prioritization among junior doctors. Its simplicity supports sustainability: it requires no additional resources, integrates easily into existing workflows, and can be adapted for use in other hospital departments. Several comparable QIPs have emphasized that sustainability depends on embedding tools into routine practice rather than reliance on individual initiative [12-14].

Looking forward, integration of the checklist into the hospital’s electronic handover system could further improve compliance tracking, streamline documentation, and enable automated audits of completeness, an approach supported by Royal College of Surgeons Safe Handover guidance [17]. Broader institutional adoption, particularly across wards with high weekend acuity, could reinforce a culture of structured, accountable handover and strengthen continuity of care across the NHS.

In summary, this project demonstrates that even small, targeted interventions can meaningfully improve the reliability and clarity of weekend handovers. The structured checklist not only addressed gaps in communication and task completion but also provided a practical, sustainable tool that fits seamlessly into existing workflows. Its low cost, simplicity, and adaptability suggest strong potential for wider implementation across hospital departments.

Limitations

While this project achieved measurable improvements, several limitations should be acknowledged. As a real-world quality improvement initiative, certain constraints were inherent to the clinical environment rather than methodological flaws. The project was conducted in a single respiratory ward with a relatively small sample size (44 handovers pre-intervention and 50 post-intervention), which limits the generalizability of the findings. Additionally, the work was not blinded, as the ward team was aware of the ongoing QIP, potentially introducing elements of observer or performance bias.

Staff engagement, particularly maintaining consistent participation from consultants and registrars, was an ongoing practical challenge, occasionally requiring reminders to complete the Friday checklist. Nonetheless, this reflects typical implementation barriers in routine clinical practice and underscores the importance of leadership support and reinforcement to embed change sustainably.

Baseline data for some parameters, such as IV fluids, nutrition, and deterioration management plans, were not collected during the initial cycle, restricting direct comparison in these domains. Moreover, the absence of formal qualitative feedback (e.g., structured staff surveys) limited exploration of perceived workflow impact and user experience.

Future work should focus on larger, multiward implementation to strengthen generalizability, incorporate formal qualitative evaluation to capture staff perspectives, and assess longer term sustainability. Embedding the checklist into electronic handover systems could facilitate ongoing audit, promote consistent use, and ensure that improvements in communication and continuity of care are sustained across the organization.

Further improvements

To further improve the quality of handover and care over the weekends, it is essential to adhere to the checklist on the Respiratory Ward. Additionally, expanding the use of the checklist to other medical and surgical specialities, as well as extending it to additional departments within the hospital, will enhance overall patient care. Incorporating the checklist into the electronic system with assistance from IT departments will ensure effective handovers to weekend teams across the NHS Trust.

## Conclusions

This quality improvement project highlights the positive impact of a structured weekend handover checklist in strengthening communication between weekday and weekend teams. By standardizing essential clinical information transfer, the intervention supported safer, more organized, and continuous patient care.

While preliminary in scope, the outcomes of this project provide encouraging evidence of the intervention’s practicality and effectiveness. Despite being conducted in a single ward with a limited sample size and brief follow-up period, the project demonstrated clear and measurable process improvements within a real-world clinical context.

As the outcomes were primarily process-based rather than patient-level, further evaluation is warranted to explore long-term clinical impact. Despite these limitations, the results align with broader literature and underscore the feasibility and sustainability of structured handover tools in enhancing communication, continuity, and patient safety.

Future work should evaluate long-term sustainability, staff engagement, and the broader effects of such interventions on patient outcomes and organizational safety culture across departments.

## Appendices

Weekend continuity checklist

**Table 2:** Weekend continuity checklist developed for the respiratory ward to standardize and improve weekend handover processes The project followed the Plan-Do-Study-Act quality improvement framework, a freely available and widely adopted model for iterative healthcare improvement [9] VTE: venous thromboembolism, TEP: treatment escalation plan, MOFD: medically optimized for discharge, EDN: electronic discharge notification, NIV: noninvasive ventilation, SHO: senior house officer

Patient name (hospital no.)	Bloods (Y/N)	Antibiotics prescribed? (Y/N)	VTE assesment? (Y/N)	Fluids if needed: plan in place?	Nutrition-specific plans (if needed) in place?	TEP/deterioration plan in place? (Y/N)	Electronic handover (if needed) in place?	If MOFD-EDN in place? (Y/N)	Respiratory-specific plans?		Weekend review? (SHO/registrar/consultant)
									If on NIV plan in place?	Chest drain in situ? Plan in place?	
1.	-	-	-	-	-	-	-	-	-	-	-
2.	-	-	-	-	-	-	-	-	-	-	-
3.	-	-	-	-	-	-	-	-	-	-	-
4.	-	-	-	-	-	-	-	-	-	-	-
5.	-	-	-	-	-	-	-	-	-	-	-
6.	-	-	-	-	-	-	-	-	-	-	-
7.	-	-	-	-	-	-	-	-	-	-	-
